# Current status of academic palliative medicine in Poland: a nationwide study

**DOI:** 10.1186/s12904-022-00983-8

**Published:** 2022-06-04

**Authors:** Wojciech Leppert, Aleksandra Sesiuk, Aleksandra Kotlińska–Lemieszek

**Affiliations:** 1 Chair of Palliative Medicine, Institute of Medical Sciences, Collegium Medicum, University of Zielona Góra, Zielona Góra, Poland; 2 University Hospital of Heliodor Święcicki, Partner of Poznań University of Medical Sciences, Poznań, Poland; 3 Department of Gastroenterology, Dietetics and Internal Medicine, University Hospital of Heliodor Święcicki, Partner of Poznań University of Medical Sciences, Poznań, Poland; 4 Laboratory of Pharmacotherapy in Palliative Care, Chair and Department of Palliative Medicine, Poznan University of Medical Sciences, Poznań, Poland

**Keywords:** Medical students, Palliative care, Palliative medicine, Undergraduate education, University

## Abstract

**Aim:**

To assess the current status of palliative medicine (PM) education in medical students in Poland.

**Methods:**

Data on PM teaching were obtained from a 16–item questionnaire sent to the heads of PM and palliative care (PC) departments at universities or university authorities. In cases in which there was no PM or PC department, the questionnaire was sent to authorities of a given University.

**Results:**

Eleven PM and PC departments were included in the analysis; 7 at the medical universities, and four at collegium medicum at universities. Among these there were two chairs of PM (at the Medical University of Poznań and the Collegium Medicum at the University of Zielona Góra) and one chair of PC (in Bydgoszcz). Most of the Departments were part of faculties of medicine, and a minority were part of faculties of health sciences. There were no PM or PC departments at 2 medical universities, three at collegium medicum at universities, and 6 at faculties of medicine; two at public universities and 4 at non–public universities. All programs of PM teaching included the philosophy of PC, and pain management. The majority included management of other symptoms, emergencies, communication, ethical issues and psychological issues in PC. Of 12 programs, 9 included practical (bedside) teaching. The numbers of hours allocated to PM ranged from 15 to 45 (median 20).

**Conclusions:**

Half of the universities that educate medical students in Poland had PM departments and provided obligatory PM teaching. Establishing departments of PM and PC at all medical universities, collegium medicum at universities, and faculties of medicine at universities with a common PM program as an integral part of undergraduate education is suggested through including PM as a separate subject to the Regulation of the Ministry of Education and Science and initiatives of National and Provincial Consultants in PM.

## Introduction

Poland currently has close to 37.8 million inhabitants, and the annual number of deaths due to chronic diseases is approximately 300,000 including nearly 100,000 deaths from cancer. Of all European countries, Poland holds the 7th place in the development of specialist palliative care [1]. Approximately 600 palliative care units operate around the country, predominantly home palliative care teams.

An overview of the status of palliative medicine tuition of medical students and palliative care tuition of nursing students as well as professorships in palliative care at public medical schools is presented in the European Association for Palliative Care (EAPC) Atlas of Palliative Medicine in Europe 2019 [1]. The incorporation of palliative care tuition (both theoretical and practical/clinical) into undergraduate nursing curricula was made obligatory by the Regulation of the Ministry of Science and Higher Education in 2019 [2]. However, palliative medicine teaching for undergraduate medical students [3–6] was not included in this Regulation, and as a consequence its content and incorporation into curricula depends on individual decisions of the authorities of medical universities, collegium medicum of universities, and faculties of medicine of public and non–public universities.

Palliative medicine education program for sixth year medical students in Poland has been developed and introduced in several medical universities in 90–ties [7, 8]. However, since that time significant progress in palliative medicine and teaching methods has been made, which should also impact education of medical students in the field of palliative medicine [9, 10]. Additionally, no formal assessment of academic units and medical students teaching in palliative medicine has been made in Poland.

A substantial number of collegium medicum and faculties of medicine were only recently established at both public and non–public universities in Poland however, thus little is known about the current situation regarding palliative medicine tuition of medical students at undergraduate level. The aim of the present study was to assess the current status of palliative medicine and palliative care academic units, and the current situation with respect to undergraduate palliative medicine education of medical students in Poland.

## Methods

A questionnaire was developed by the authors aimed at gaining knowledge on academic palliative medicine teaching. The questionnaire consisted of 16 questions and a space provided for free comments of respondents who filled the questionnaire.

The first two questions have yes (no) answers if there is an obligatory education on palliative medicine (palliative care) and if there is a separate course on palliative medicine or if palliative medicine is included in another subject and if so, what subject. Two questions referred to the name of the unit and a teaching coordinator. Another question referred to faculties at which classes are held (medicine, nursing, paramedics, physiotherapy, dietetics and other). Next questions referred to number of hours allocated, if they increase, decrease or remained the same as in former academic year, form of classes (lectures, seminars, classes without and with patients), a proportion (in percentage) of theoretical and practical classes, method of getting credit, number of teachers involved and their academic degrees. The last four questions referred to if there are optional courses in palliative medicine (or selected topics of palliative medicine) and if yes what is a title of the subject and a number of hours allocated, manuals and other didactic materials provided to students, and a last question – a person who filled in this questionnaire with an e–mail address. There was a space provided for free additional comments.

The questionnaire was sent electronically to heads of academic palliative medicine and palliative care departments or coordinators of palliative medicine teaching at Polish universities where education is provided to medical students. In cases in which there was no palliative medicine or palliative care department, the questionnaire was sent to the authorities of a given university. Questions that were not clear or certain were discussed during direct phone calls or additional e–mail communication. All data analyzed in this study refer to an academic year 2019/2020.

## Results

The questionnaire response rate was 100%.

### Academic units of palliative medicine

Currently there are 11 academic palliative medicine and palliative care departments in Poland; 7 at the Medical Universities of Białystok, Gdańsk, Katowice, Łódź, Poznań, Warsaw (Warszawa), and Wrocław, and 4 at collegium medicum at the Universities of Bydgoszcz, Kraków, Olsztyn, and Zielona Góra. At the time of the current study, of these 11 Departments there were 2 chairs of palliative medicine (at Poznan University of Medical Sciences in Poznań and the Collegium Medicum at the University of Zielona Góra – both affiliated with a faculty of medicine) and 1 chair of palliative care (at Ludwik Rydygier Collegium Medicum in Bydgoszcz, Nicolaus Copernicus University in Toruń – affiliated with a faculty of health sciences).

Of the remaining 8 departments of palliative medicine and palliative care, 6 were part of faculties of medicine (Medical Universities of Białystok, Gdańsk, Łódź, and Warsaw, and Collegium Medicum at Jagiellonian University in Kraków, and the University of Warmia and Mazury in Olsztyn) and 2 were attached to faculties of health sciences (Medical University of Silesia in Katowice and Wrocław Medical University). Of the 8 aforementioned departments, 6 are part of other chairs; Chair of Oncology at the Medical University of Łódź (Department of Palliative Medicine), and the Collegium Medicum at the University of Warmia and Mazury in Olsztyn (Laboratory of Palliative Medicine and Psychooncology), Chair of Family Medicine at the Medical University of Gdańsk (Department of Palliative Medicine), Chair of Geriatrics and Internal Medicine at Jagiellonian University Collegium Medicum in Kraków (Department of Pain and Palliative Care), Chair of Nursing at the Medical University of Silesia in Katowice (Department of Palliative Medicine and Care), and Chair of Clinical Nursing at Wroclaw Medical University (Department of Oncology and Palliative Care). The Laboratory of Palliative Medicine at the Medical University of Warsaw is linked to the Department of Society Medicine and Public Health, and the Department of Palliative Medicine at the Medical University of Białystok is an independent academic unit. There were no palliative medicine or palliative care departments at 11 universities, including 2 medical universities, 3 collegium medicum at universities, and 6 faculties of medicine (2 at public universities and 4 at non–public universities).

### Teaching palliative medicine at universities

Palliative medicine was taught at 12 universities, including 7/9 medical universities and 5/7 collegium medicum at universities. In all of them, it was an obligatory and separate course, with the exception of non–compulsory classes at the Collegium Medicum at the University of Rzeszów. Palliative medicine was not taught at 10 universities, including 2 medical universities, 2 collegium medicum at universities, and 6 faculties of medicine (2 at public universities and 4 at non–public universities). Only 3 new programs were introduced in the last decade. At 9 universities, palliative medicine was included in the 5th–year curriculum, at 4 it was taught in the 4th year, at 2 it was taught in the 6th year, and in 1 it was taught in the 3rd year. At 3 universities, palliative medicine was included in the curricula of 2 or 3 subsequent years.

### Palliative medicine courses: content, teaching methods, duration and getting credit

**Fig. 1:**
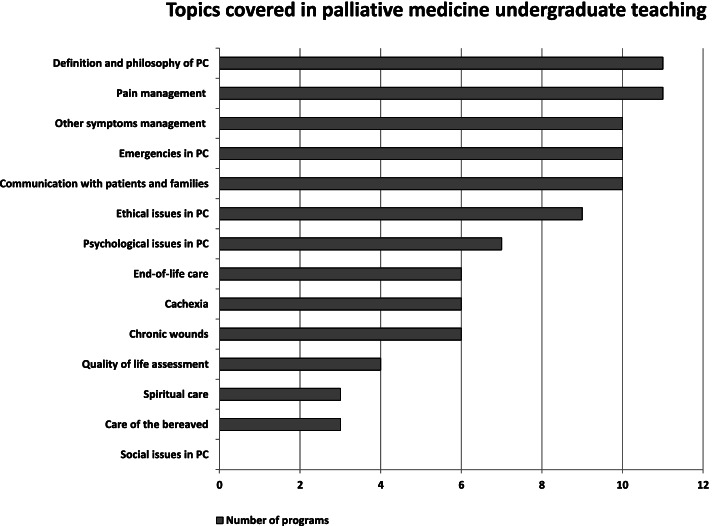
Topics covered during palliative medicine tuition of medical students (11 obligatory curricula included)

All mandatory curricula covered topics related to the philosophy of palliative care as well as pain management. The vast majority of programs included management of symptoms other than pain, emergencies in palliative care, communication, and ethical issues. More than half of the curricula included psychological issues related to palliative care, end-of-life care, cachexia, and the prevention and management of chronic wounds. Quality of life was incorporated as a separate topic in 4/11 curricula, and spiritual care and issues related to bereavement were included in 3 programs. None of the programs included separate hours devoted to social issues in palliative care (Fig. 1).

**Fig. 2:**
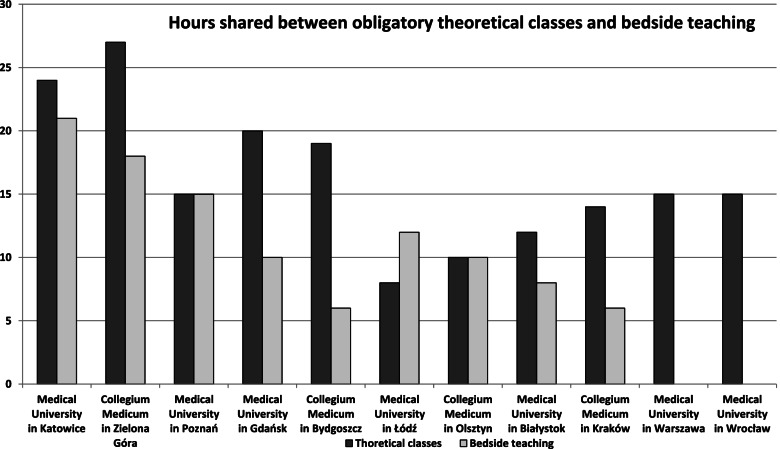
Numbers of hours allocated to theoretical and practical (bedside) teaching of palliative medicine to medical students

Teaching formats included lectures, seminars, workshops for smaller groups, and case presentations prepared by students. Practical (bedside) teaching sessions involving patients, which were provided at 9 academic palliative medicine and palliative care departments, were conducted at in–patient palliative medicine units or at stationary hospices and out–patient palliative medicine clinics (Fig. 2).

There were substantial differences in the numbers of hours allocated to palliative medicine teaching at different universities (mean 26 h, median 20 h, range 15–45 h). The number of hours exceeded 40 only in two departments of palliative medicine. In two departments, the number of hours had increased in recent years but remained low (15 h and 20 h). In another two departments, the numbers of hours had been reduced to 20 h and 30 h in recent years. In the majority of academic departments of palliative medicine, the students satisfied the relevant requirements of the course via a multiple choice test, and in some programs, this was supplemented with case presentations.

### Multidisciplinary palliative medicine teaching team

**Fig. 3:**
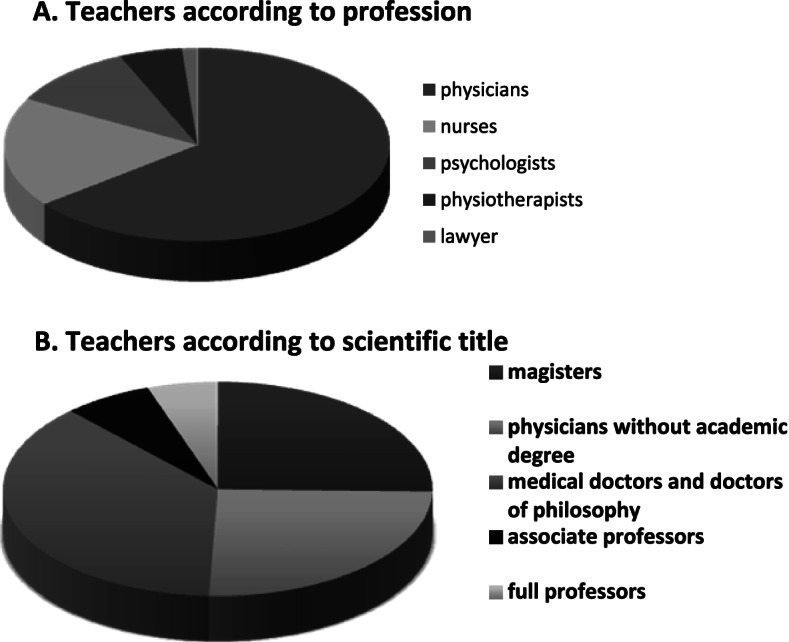
Teachers involved in palliative medicine education of medical students according to **A** profession and **B** scientific title

In 10 departments of palliative medicine and palliative care, physician specialists in palliative medicine, specialists in palliative care nursing, clinical psychologists, physiotherapists, and a lawyer (at one department) were involved in medical students’ education (Fig. 3A). Approximately half of all teachers possessed scientific titles such as a medical doctorate or a PhD. There were 4 full professors and 5 associate professors responsible for teaching palliative medicine at 8 academic palliative medicine departments (Fig. 3B).

## Discussion

This questionnaire–based study aimed to assess the current status of academic palliative medicine and palliative care units, and the current level of undergraduate education in palliative medicine delivered to medical students in Poland. Undergraduate palliative care education of nursing students and palliative medicine education of medical students have major impacts on the development of palliative care and palliative medicine.

### Academic palliative medicine units

In Poland, faculties of medicine that provide undergraduate education to medical students were present at 22 universities, including 9 medical universities, 7 collegium medicum at universities, and 6 faculties of medicine (2 at public universities and 4 at non–public universities). At the time of the current study, academic palliative medicine units were present at 11/22 universities (50%) that provide education to medical students. There were half of universities that did not possess academic palliative medicine units. There is a need to establish academic palliative medicine departments at all universities that provide teaching at faculties of medicine. Preferably, chairs should provide leading academic positions (professorships) that may better fulfill requirements of research development and undergraduate and postgraduate education needs [4]. This may be achieved by introducing national standards, which should be obligatory for all faculties of medicine after a transitional period for organization and building of multidisciplinary teams of academics and clinicians.

### Teaching palliative medicine at universities

The present study identified a lack of obligatory palliative medicine teaching at 11/22 universities that possess faculties of medicine and provide undergraduate education to medical students. This is most probably caused by a lack of palliative medicine academic units and qualified teachers. This situation is concordant with recent data from some other European countries [1, 3]. It should be also taken into consideration that there are a substantial number of new universities, medical colleges and faculties of medicine in Poland that were established 2–5 years ago and are in the process of early development, and students are in the first years of their studies. Because palliative medicine is predominately included in the final (typically 5^th^) year curricula, these students were not taught palliative medicine.

Palliative medicine curricula introduced into medical studies in Poland were formulated by teaching teams, and there was substantial heterogeneity of content. All programs included the philosophy of palliative care and pain management, the vast majority included the management of other symptoms, emergencies, communication, ethics and more than half psychological issues in palliative care [11]. This is consistent with recommendations of the EAPC, which suggest that 6 main domains be included in the syllabus; philosophy, general characteristics and organization of palliative care, pain and symptom management, psychosocial and spiritual aspects, ethical and legal issues, communication and teamwork, and self–reflection [5]. Only the latter is not included in the majority of programs, though it may be incorporated into the philosophy of palliative care during introductory lectures as reported by some palliative medicine coordinators. Interestingly, in 10/11 syllabuses the topic “emergencies in palliative care” was also included. These issues overlap with symptom management, and their inclusion in the syllabus may reflect an aim of preparing students to diagnose and manage life–threatening clinical conditions that usually cause severe suffering.

Not all programs included practical (bedside) teaching, which should be obligatory in medical students’ education. This situation results from a lack of in–patient palliative medicine units at many university hospitals, and their establishment at all medical universities and colleges of medicine is highly recommended.

Notably all but 2 existing programs used a multidisciplinary team of teachers with different backgrounds, including physicians, nurses, and psychologists. This is more likely to teach medical students core competencies in palliative care, and should be regarded as mandatory [6]. The number of academic teachers and teaching coordinators with professorships was comparable to other European countries [1].

In Poland, the first palliative medicine program for medical students and palliative care program for nursing students were introduced by the late Professor Jacek Łuczak and his co–workers at Poznan University of Medical Sciences in 1991 [7, 8]. This was a pioneering initiative and they were among the first undergraduate palliative medicine and palliative care programs in the world [10]. Soon thereafter several universities in Poland introduced palliative medicine into their undergraduate medical student programs [8]. Despite early initiatives, palliative medicine is still not taught to a substantial number of medical students in Poland. In the last decade, only 3 new programs were introduced.

The numbers of hours allocated to palliative medicine teaching at most academic units was low (median 20 h), and only two departments reported 40 h as recommended by the EAPC [5]. However, the numbers of hours were not lower than those reported in some other countries [4, 12]. The reason for the low numbers of hours allocated to palliative medicine may be related to a lack of inclusion of palliative medicine as a separate subject in a Ministry of Health Decree [2]. It may also be that the importance of palliative medicine is not fully appreciated by authorities of universities, and there is competition for time within the undergraduate curricula [13]. Another problem is a lack of palliative medicine specialists willing to be involved in education and research. The EAPC recommends the allocation of hours devoted to palliative care in different years, with the basics being taught as early as possible and clinical aspects being taught later in the medical course (the concept of vertical integration) [5]. In Poland, three departments of palliative medicine provided classes within more than one year of the course.

The World Health Organization has stated that “Education on palliative care should be integrated as a routine element of all undergraduate medical and nursing professional education” [14]. According to the EAPC White Paper on Palliative Care Education – Part 1, “all healthcare professionals and workers should be able to provide appropriate palliative care and thus need to be trained to provide the highest possible standards of care in order to meet the challenging needs of patients and families” [15]. Recommendations concerning palliative medicine teaching at medical schools on a national level were implemented in some countries (for example Germany), and catalyzed the integration of palliative medicine into core medical education [16]. Studies on palliative care teaching for medical students reveal significant variation between countries and within countries, but generally demonstrate improvement in the last two decades [1, 3, 4, 12, 13, 16–18]. The lack or insufficiency of undergraduate and postgraduate medical student education in palliative medicine is one of the biggest barriers to the development of palliative care.

The main limitation of the current study was that the evaluation was based on a questionnaire and personal communications only. Also, the study refers to academic year 2019/2020 and currently efforts are made to introduce palliative medicine teaching for medical students at two medical universities and two collegium medicum at universities. To the best of our knowledge however, it is the first study to assess the current status of academic palliative medicine in Poland.

## Conclusions

Only half of the universities that educate medical students in Poland had palliative medicine departments and provided obligatory palliative medicine teaching. There were substantial differences in the numbers of hours allocated to palliative medicine and the curricula within different faculties of medicine. Moreover, not all academic units of palliative medicine provided clinical (bedside) teaching.

The present situation needs improvement in order to develop better undergraduate palliative medicine education for all medical students in Poland. This may be achieved by establishing academic units of palliative medicine at all universities that possess faculties of medicine, as well as the introduction and development of a common palliative medicine curriculum as an integral part of undergraduate education for medical students, including practical classes with patients.

Establishing departments of palliative medicine and palliative care at all medical universities, collegium medicum at universities, and faculties of medicine at universities with a common palliative medicine program as an integral part of undergraduate education is strongly recommended. It may be achieved through including palliative medicine as a separate subject to the Regulation of the Ministry of Education and Science and initiatives of National and Provincial Consultants in palliative medicine.

## Data Availability

All data are available on request to Wojciech Leppert e–mail: wojciechleppert@wp.pl.
